# Role of tryptophan-metabolizing microbiota in mice diarrhea caused by *Folium sennae* extracts

**DOI:** 10.1186/s12866-020-01864-x

**Published:** 2020-06-29

**Authors:** Chenyang Zhang, Haoqing Shao, Dandan Li, Nenqun Xiao, Zhoujin Tan

**Affiliations:** 1 School of Traditional Chinese Medicine, Hunan University of Chinese Medicine, Changsha, Hunan China; 2 Hunan Key Laboratory of TCM Prescription and Syndromes Translational Medicine, Changsha, Hunan China; 3 Hunan University of Chinese Medicine, Changsha, Hunan China

**Keywords:** Diarrhea, Intestinal microbiota, Cytochrome P450, Tryptophan metabolism, *Folium sennae*

## Abstract

**Background:**

Although reports have provided evidence that diarrhea caused by *Folium sennae* can result in intestinal microbiota diversity disorder, the intestinal bacterial characteristic and specific mechanism are still unknown. The objective of our study was to investigate the mechanism of diarrhea caused by *Folium sennae*, which was associated with intestinal bacterial characteristic reshaping and metabolic abnormality.

**Results:**

For the intervention of *Folium sennae* extracts, Chao1 index and Shannon index were statistical decreased. The Beta diversity clusters of mice interfered by *Folium sennae* extracts were distinctly separated from control group. Combining PPI network analysis, cytochrome P450 enzymes metabolism was the main signaling pathway of diarrhea caused by *Folium sennae*. Moreover, 10 bacterial flora communities had statistical significant difference with *Folium sennae* intervention: the abundance of *Paraprevotella*, *Streptococcus*, *Epulopiscium, Sutterella* and *Mycoplasma* increased significantly; and the abundance of *Adlercreutzia, Lactobacillus*, *Dehalobacterium*, *Dorea* and *Oscillospira* reduced significantly. Seven of the 10 intestinal microbiota communities were related to the synthesis of tryptophan derivatives, which affected the transformation of aminotryptophan into L-tryptophan, leading to abnormal tryptophan metabolism in the host.

**Conclusions:**

*Folium sennae* targeted cytochrome P450 3A4 to alter intestinal bacterial characteristic and intervene the tryptophan metabolism of intestinal microbiota, such as *Streptococcus*, *Sutterella* and *Dorea*, which could be the intestinal microecological mechanism of diarrhea caused by *Folium sennae* extracts.

## Background

Diarrhea is one of the common side effects of drugs [1], and may cause other diseases [2]. *Folium sennae* (leaves of *Cassia angustifolia* or *Cassia senna*) is a laxative, and a traditional Chinese purgative medicine that can cause diarrhea as well, commonly used for the treatment of diet, stress, or medication related constipation [3]. Nowadays, for its diarrhea-causing function *Folium sennae* is very often used to construct animal models to support modern research on diarrhea-related diseases [4, 5].

Modern pharmacological studies have shown that pharmacological and toxicological effects of *Folium sennae* are inseparable. It is generally accepted that a variety of anthranoids are the major contributor of diarrhea caused by *Folium sennae* such as sennosides, aloe-emodin, rhein, and chrysophanol [6]. Anthraquinones possessed a wide of pharmacological including laxative, anti-tumor, anti-bacterial, anti-inflammatory and other activities, but also led to hepatotoxicity, renal toxicity and genetic damage in high doses [6, 7]. The European Medicines Agency Revised Draft Assessment Report and Herb on Senna Folium and Senna Fructus recommended that the dosage and duration of clinical application of *Folium sennae* need to be limited for its adverse effects of long-term abuse and potential carcinogenicity. As far as the future in-depth research of pharmacological activity, clinical efficacy and safety, there represents a challenge to isolate the new potential chemical compounds with suggestive classes [8]. Besides, animal experiments have suggested that *Folium sennae* constituents caused liver injury in rats through metabolism disorder [6]; and serum adrenocorticotropic hormone and 24 h urine 17 - hydroxycorticosteroid were significantly reduced in rats with a high concentration and long-term gastric administration of a senna leaf decoction [9]. Even though experimental evidence supported diarrhea caused by *Folium sennae* could affect multiple systems of an organism, and what is more the growth of microorganisms [6], the pharmacological activities is largely unknown so far.

Under most conditions, changes of intestinal microecology including microbes and the metabolic products are closely connected to diarrhea. The decreased diversity of intestinal microbiota has been proved in diarrhea caused by *Folium sennae* model of KM mice [10]. Moreover, gut microbiome-host interactions extend beyond the digestion of food and nutrient absorption and have widespread biological implications [11]. More and more evidence showed intestinal microecology plays a significant role in the health of the host [12]; and gut microbiome perturbations and microbial metabolites dysbiosis have been implicated in the progression and pathogenesis of hepatopathy [13], nephropathy [14], cardiovascular disease [15], etc. Metabolism plays a crucial role in human health and disease, and it is modulated by intrinsic (e.g. genetic) and extrinsic (e.g. diet and gut microbiota) factors [16, 17]. Thus, considering the effect of *Folium sennae* on the multiple systems of an organism, there are reasons to believe that the changes of intestinal microbiota and microbial metabolites may be a way of pharmacological mechanism of diarrhea induced by *Folium sennae*. A broad spectrum of disease with intestinal microbiota disorders [13], whether the change of intestinal microbiota characteristics is the cause of diarrhea-related diseases needs to be further studied.

This study consisted of the following 3 steps. We started with evaluating the effects of *Folium sennae* on the change of intestinal microbiota characteristics by using long-term diarrhea model constructed with *Folium sennae*. Next, we screened the active compounds of *Folium sennae* and predicted their diarrhea-related targets and metabolic pathways. And then, we used Virtual Metabolic Human (VMH) to simulate metabolic function of bacteria associated with senna-induced diarrhea to verify the metabolic pathways. Ultimately, we found that *Folium sennae* affected tryptophan metabolism of intestinal microbiota and thus diarrhea disease, in which CYP3A4 may be the key target.

## Results

### Praxiology change of mice with stomach perfusion of *Folium sennae* extracts

During gastric administration with sterile saline, the mice in pfck group had dense and glossy back hair with bright eyes; a diet water quantity, stools and mental state were normal. Diarrhea symptoms appeared in the afternoon of the first day of *Folium sennae* extracts given by gavage in pfm group mice, and feces began to become thinner and softer. Feces of pfm group mice became thinner and perianal contamination on the second day of modeling. Mice in pfm group began to show hairy back, sleepiness, arched back and dry back; and diarrhea symptoms in the afternoon were more serious than those in the morning of the third day of modeling. On the 8th day, it was found that the stool in pfck group was water-like after dissecting the mice, while in pfm group, the stool was granular, moist, dark, normal in size and shape. There was no significant difference in body weight change and body weight change rate between pfm group and pfck group [(9.97 + 4.48) g vs (10.26 + 5.00) g, *p*-value = 0.06; (29.23 + 9.87) % vs (28.74 + 10.86) %, *p*-value = 0.06].

### Intestinal microbial diversity in diarrhea mice caused by *Folium sennae* extracts

#### Sample sequences and operational taxonomic units

The number of 16S rRNA PCR sample sequences is positively correlated with the number of microbial populations, and the intensity of sample sequences indicates the number of DNA fragments, hence the size of a certain microbial population. There were a total of 475,973 effective sequences and 439,518 high quality sequences obtained by sequencing mostly between 150 bp and 300 bp in this study for subsequent analysis.

**Fig. 1:**
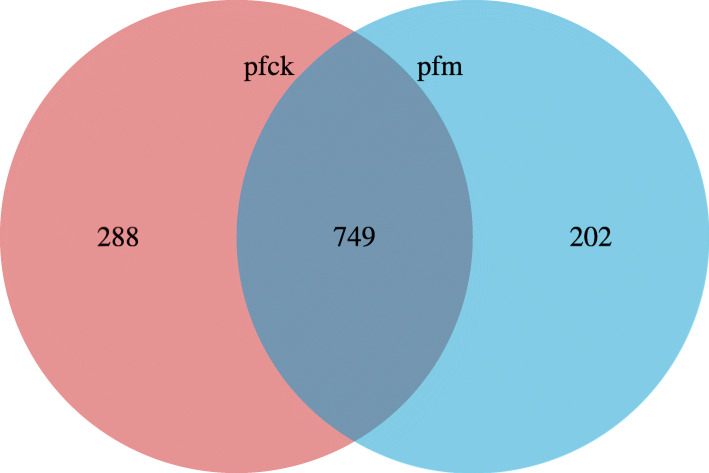
Venn diagram of the distribution of OTUs in the 2 groups

According to sequence homogeneity, Qiime soft was used in clustering 97% similarity DNA sequences into a same Operational Taxonomic Unit (OTU). As shown in Fig. 1, 288 and 202 OTUs were uniquely identified from two groups, respectively; and the number of OTUs coincided between the two groups was 749. The result suggested that the OTU of bacterial gene had decreased after diarrhea caused by *Folium sennae*.

#### Bacterial diversity in intestinal contents of diarrhea mice caused by *Folium sennae*

**Fig. 2:**
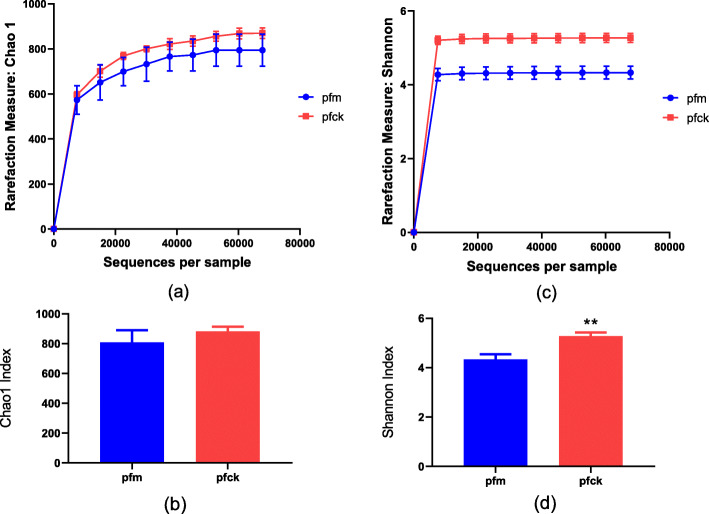
Alpha diversity comparison of bacterial genes in intestinal contents between pfck and pfm. **a** Comparison of Chao1 index between pfck and pfm and Chao1 index curve under various sequencing lengths; **b** total difference of Chao1 index between the two groups; **c** Comparison of Shannon index between pfck and pfm and Shannon index curve under various sequencing lengths; **d** total difference of Shannon index between the two groups. **, *p*-value< 0.01

In order to illustrate the diversity of intestinal microbiota in the two group of mice, we calculated Alpha diversity and Beta diversity [18]. The Alpha diversity index includes Chao1 (showing the richness of bacteria) and Shannon (showing the diversity of bacteria): the higher the Alpha diversity, the better the richness of the bacterial species, the more uniform the number of intestinal bacteria, and the more stable the microbiota. The results showed that both the Chao1 index curve (Fig. 2a) and Shannon index curve (Fig. 2c) of the sample had entered the plateau and reached saturation, which suggested that the amount of sequencing data of our study is large enough to reflect the vast majority of microbial species information in the sample. As shown in Fig. 2, within all the range of gene sequences pfm group mice showed lower Chao1 index and Shannon index. Overall, Chao1 index (Fig. 2b) and Shannon index (Fig. 2d) decreased with no statistically significant after *Folium sennae* intervention, and Shannon index decreased with statistical significance (*p*-value < 0.05). It suggested that *Folium sennae* could affect intestinal microbiota diversity in mice.

**Fig. 3:**
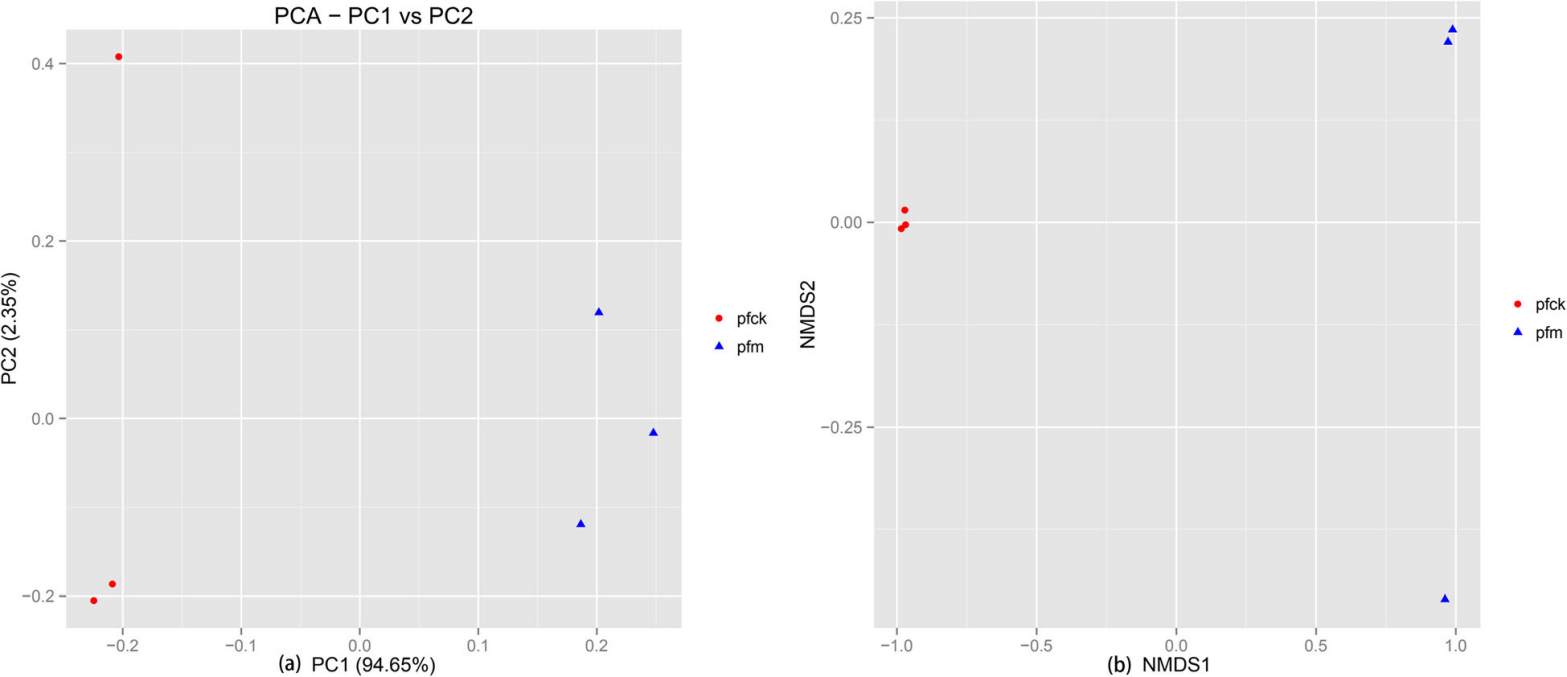
Beta diversity comparison of bacterial genes in intestinal contents between pfck and pfm. **a** Principal Component Analysis (PCA); the dominant bacterial communities of the gut microbiota in the 2 groups; the weights of PC1 and PC2 are 0.97, and amount of information contained is sufficient to illustrate the overall sample situation; **b** Analytical diagram of NMDS; each point in the figure represents a sample; the points with the same color belong to the same group; the closer the distance between the two points, the smaller the difference of microbial communities between the two samples

Beta diversity is a comparative analysis of microbial community composition of different samples. PCA and NMDS analysis was conducted to measure differences in communities of bacteria at genus level to illustrate the Beta diversity. PCA of log2-transformed normalized abundance for OTUs with normalized total abundance. Unifrac analysis was carried out to obtain the distance matrix of the differences between samples by using the evolutionary information of species and the abundance information of species. The distance matrix information between the samples was analyzed by NMDS. As shown in Fig. 3, with PCA (Fig. 3a) or NMDS (Fig. 3b) analysis of OTU, OTUs of pfm group were mainly concentrated in the right quadrant. Thus it can be inferred that the intestinal microbial diversity of normal mice could be changed by *Folium sennae*.

### Active ingredients of *Folium Sennae* regulate the key protein CYP3A4 and influence tryptophan metabolic pathways associated with diarrhea

#### Filtration of active ingredients and potential target genes associated with diarrhea of *Folium sennae*

Total 53 chemical constituents of *Folium sennae* were retrieved from TCMSP. All compounds were subjected to Active Perl 5.26 screening, and a total of 10 active compounds had OB ≥ 30% and DL ≥ 0.18. Therefore, the selected 10 compounds (Table 1) from *Folium sennae* were subjected to further analysis. After removing the redundancy, total of 370 potential targets (**Table S****1**) were obtained from the 10 active ingredients. Genecard database was used to establish a new diarrhea-related gene database, which was docked with the selected 370 target genes of *Folium sennae*. Finally, 51 potential target genes of *Folium sennae*-Diarrhea were filtered out (**Table S****2**).

**Table 1:** The selected 10 compounds from *Folium sennae*

Molecule ID	Molecule Name	Molecular Structure	OB% ^a^	DL ^b^
MOL002259	Physciondiglucoside		41.65	0.63
MOL002268	rhein		47.07	0.28
MOL002276	Sennoside E_qt		50.69	0.61
MOL002288	Emodin-1-O-beta-D-glucopyranoside		44.81	0.80
MOL002293	Sennoside D_qt		61.06	0.61
MOL002369	Dihydroxydianthrone		74.55	0.57
MOL002372	(6Z,10E,14E,18E)-2,6,10,15,19,23-hexamethyltetracosa-2,6,10,14,18,22-hexaene		33.55	0.42
MOL000359	sitosterol		36.91	0.75
MOL000422	kaempferol		41.88	0.24
MOL000449	Stigmasterol		43.83	0.76

^a^ oral bioavailability; ^b^ drug-likeness

#### Compounds-Targets-Disease (C-T-D) network construction and analysis

**Fig. 4:**
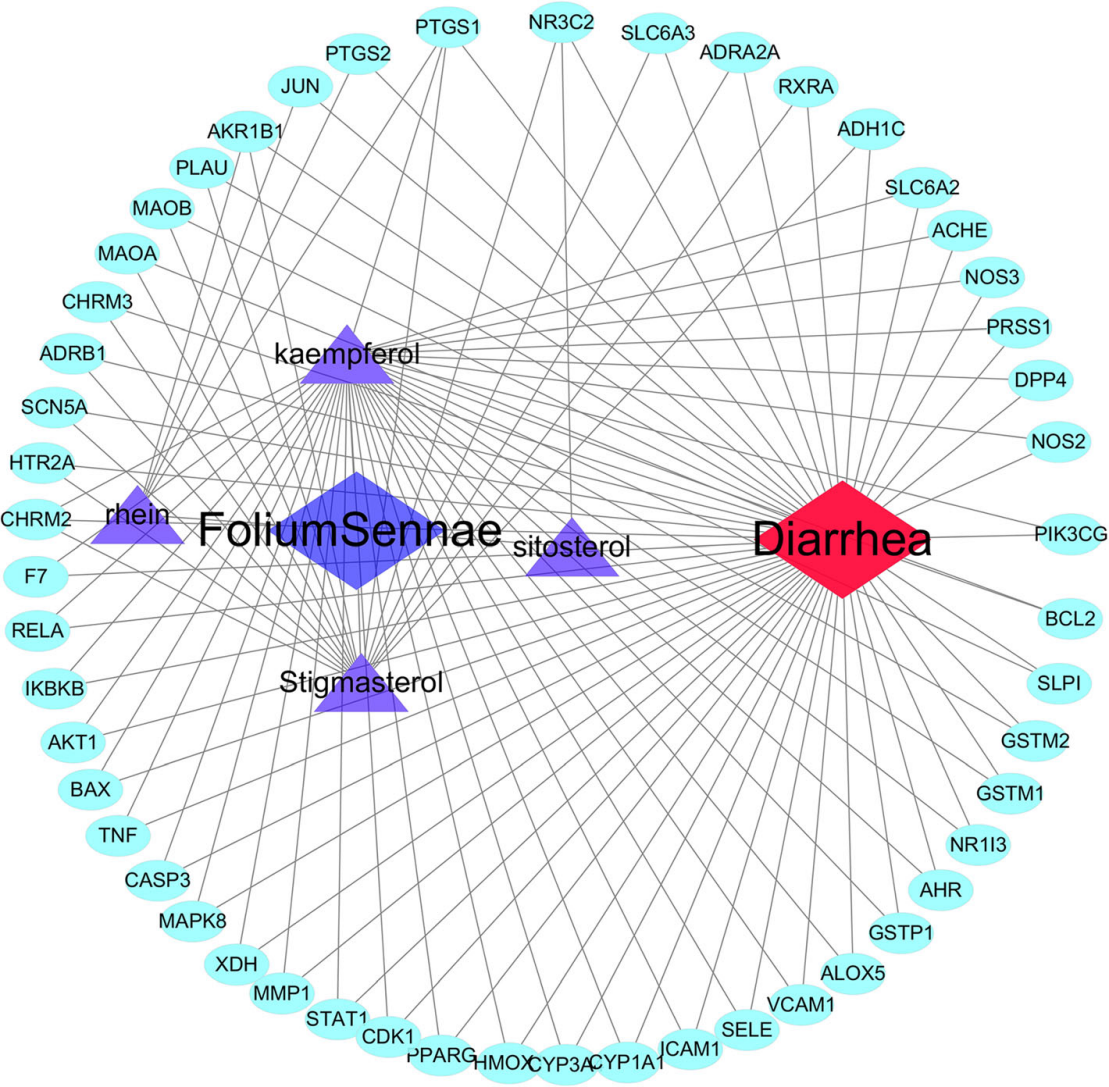
Potential active compound-target network of *Folium sennae* acting on diarrhea (C-T-D network). A compound node and a target node are linked if the protein is targeted by the related compounds; purple rhombic nodes, represent the active compounds of *Folium sennae*; blue elliptic nodes are the potential targets of *Folium sennae*; the red node represents the disease

After removing the redundancy lacking target protein gene information, we further eliminated compounds whose target genes did not intersect with the diarrhea-related gene database. A total of 4 compounds were incorporated into the C-T-D network construction, including rhein, kaempferol, sitosterol and stigmasterol. As shown in Fig. 4, the 4 active compounds and related 51 target genes constructed the network schematic diagram. Totally, this C-T-D network is composed of 57 nodes (4 active compounds and 51 potential targets) and 333 edges. In this picture, the edges indicated an association between the active ingredients, target genes and diarrhea. Degree values indicated the intensity of the interaction among the ingredients of *Folium sennae*, target genes and diarrhea, particularly in the C-T-D network kaempferol was the potential active ingredient that could regulate the CYP3A4 protein target gene, which had an effect on diarrhea.

#### PPI network construction and analysis

**Fig. 5:**
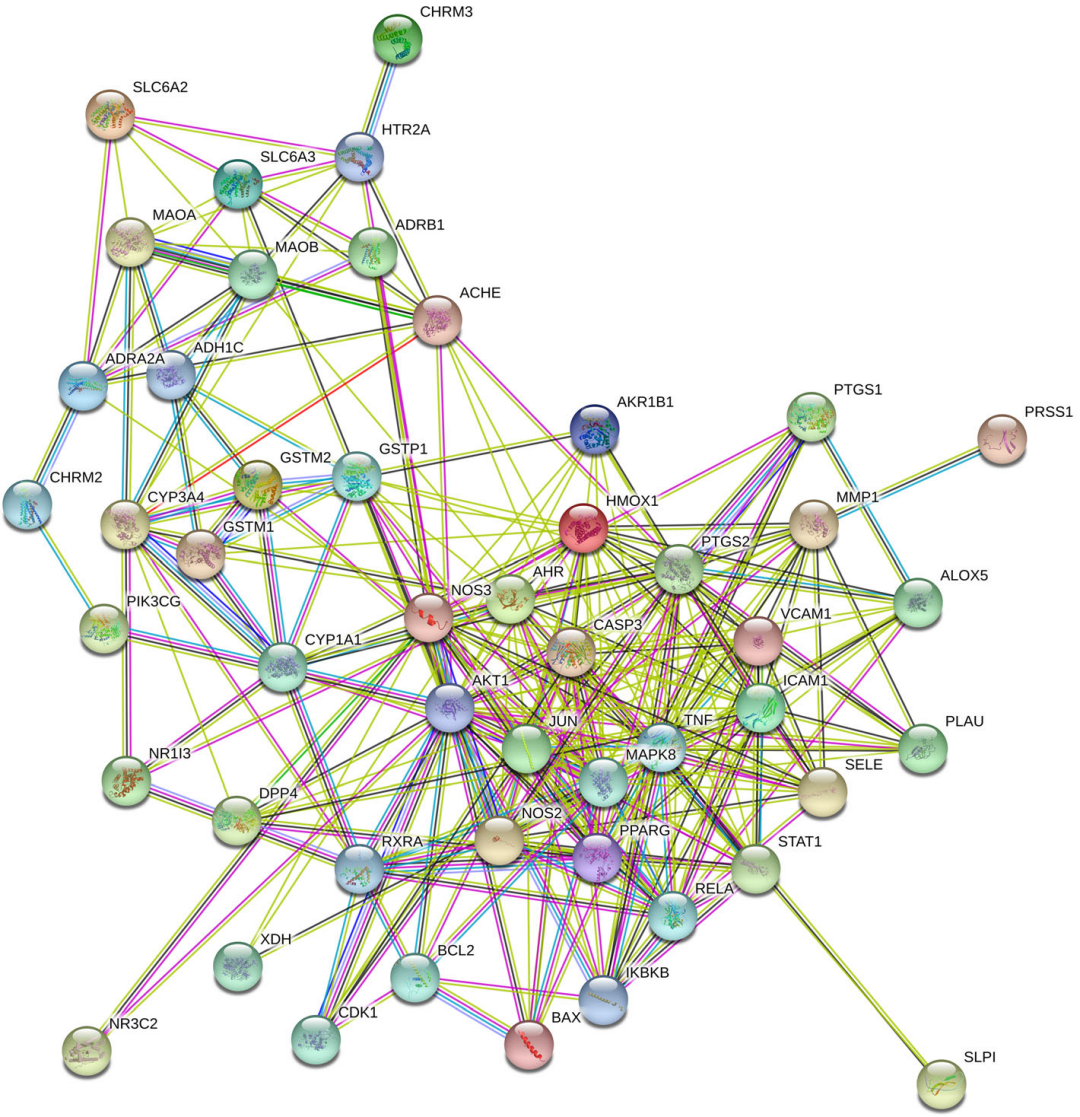
PPI network analysis of diarrhea disease targets of *Folium sennae*. Network nodes represent proteins; edges represent protein-protein associations; edges color indicates type of interaction evidence; nodes color indicates the groups that genes clustered

We constructed functional protein association PPI network to screen target protein genes that play a key role in diarrhea caused by *Folium sennae*. Forthy-nine target genes (2 disconnected nodes of genes, F7 and SCN5A, were hid) associated with active ingredients and diarrhea were imported into the STRING database for PPI network construction and analysis. There are 49 interacting targets in the network, resulting in 278 edges representing the interaction between proteins. As shown in Fig. 5, the 49 protein genes were clustered into three groups according to the interaction relationship, showing three different node colors; in the PPI network, the line color indicates 7 different type of interaction evidence: gene neighborhood, gene fusions, gene co-occurrence, co-expression, protein homology, texting and experimented.

**Fig. 6:**
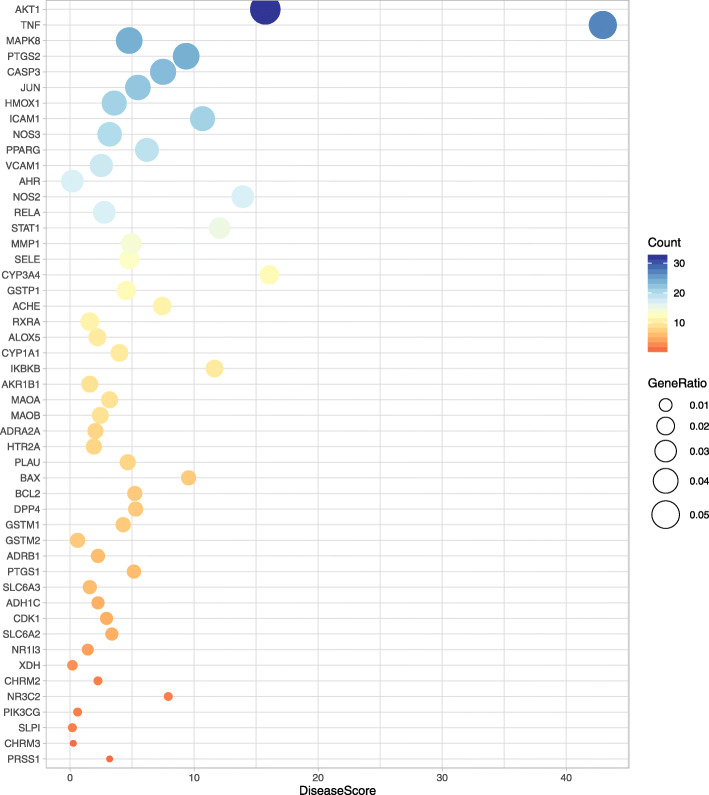
Filtration of diarrhea targets by *Folium sennae*. Disease Score, represents the relationship between target and disease; color and size of the node, represent the count and the ratio of connections between target proteins in the PPI network

According to the protein relationship of PPI network, the protein relationship is further analyzed by the number of protein links. Combining the disease score of genes (the higher the score, the more evidence is available for disease regulation), and the disease score originates in the diarrhea related gene database (**Table S****2**), the key proteins are selected. As shown in Fig. 6, the color of protein dots represents the counting of PPI network protein connections. The horizontal ordinate represents the disease score of genes, and there are three protein genes include AKT1, TNF and CYP3A4 with disease score greater than 15. The higher the disease score represents the stronger the evidence of diarrhea related protein genes, and AKT1, TNF and CYP3A4 are the main target protein genes of diarrhea caused by *Folium sennae*.

#### Kyoto encyclopedia of genes and genomes (KEGG) metabolic signaling pathway analysis

**Fig. 7:**
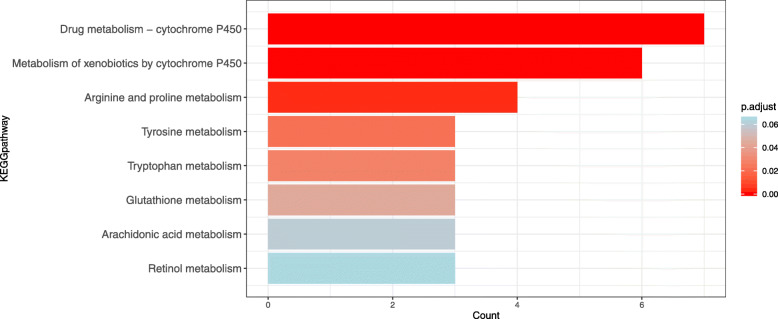
KEGG metabolic pathway enrichment analysis for core targets of *Folium sennae* acting on diarrhea

Intestinal microbiota can synthesize vitamins and amino acids necessary for human growth and development through bacterial metabolism, and it participates in the metabolism of sugars and proteins [11]. Both metabolism of substance and energy in human body by intestinal microbial community and metabolites of intestinal microbiota community play an important role. In order to effectively connect with the microbiota function, we choose the perspective of metabolic pathways to explore the mechanism of diarrhea caused by *Folium sennae* and intestinal microbiota disorder. To determine the relevant metabolic signaling pathways involved in diarrhea effect of *Folium sennae* and intestinal microbiota, we conducted pathway enrichment analysis using KEGG metabolic pathways. A total of 51 targets obtained 80 KEGG signaling pathways, and 57 channels were significantly enriched (*p*-value < 0.05). As shown in Fig. 7, among the 80 KEGG signaling pathways there were 8 KEGG metabolic signaling pathways of gene enrichment. The color of the bars in the graph was decided the *p*-values, and there were statistically significant the first 6 metabolic signaling pathways (*p*-value < 0.05). The abscissa indicates the count of proteins enriched in the pathway. The pathway enriched more protein, the more evidence suggests that this metabolic pathway is the main mechanism of diarrhea caused by *Folium sennae*. Drug metabolism - cytochrome P450 (CYP450) and metabolism of xenobiotics by CYP450 are the top 2 metabolic pathways of diarrhea caused by *Folium sennae* with count 7 and 6 respectively. In addition, the results of this study suggested that arginine and proline, tyrosine and tryptophan metabolism are the main pathways of diarrhea caused by *Folium sennae*.

Previous studies have suggested that the amino acid tryptophan played a key role in the agonistic binding of an inducer of CYP450 by activation of the constitutive androstane receptor and confirmed the structural impact of mutations of tryptophan on CYP450 [19, 20]. Therefore, we hypothesized that diarrhea caused by *Folium sennae* could affect tryptophan metabolism by diversity disorder of intestinal microbiota. Based on the sequencing results of microbial taxonomy, we validated the hypothesis by simulating intestinal microbial metabolic activities with VHM.

### Diarrhea caused by *Folium sennae* affects intestinal bacterial characteristic and tryptophan metabolism

#### Diarrhea caused by *Folium sennae* extracts affects intestinal bacterial characteristic in mice

**Fig. 8:**
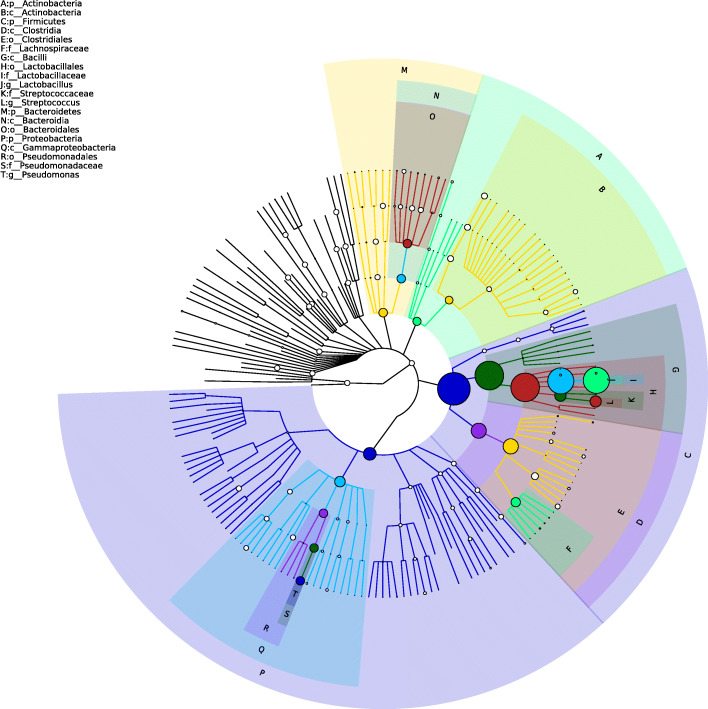
Community structure map of mice intestinal microbiota of the two groups at the genus level. The size of the node reflects the species abundance at the corresponding species level, and the top 10 genus levels are identified in the graph

According to the OTU annotation results, the abundance level and composition ratio of each sample in different taxonomic levels had been obtained, which reflects the community structure of pfck group and pfm group in different taxonomic levels. By comparing the community structure of the two groups in different taxonomic levels, the effects of *Folium sennae* on the community structure of intestinal microbiota in mice were analyzed. In this study, abundance of bacteria of OTU in genus level of single sample in pfck group and pfm group was obtained. Based on the information of microbiota and its abundance, the structure map of microbiota community could reflect comprehensively the distribution of microbiota and abundance in samples, and the microbiota with higher abundance (top 20) could be found (Fig. 8). The picture had reflected the overall difference in the intestinal bacterial characteristic of the two groups of mice.

**Fig. 9:**
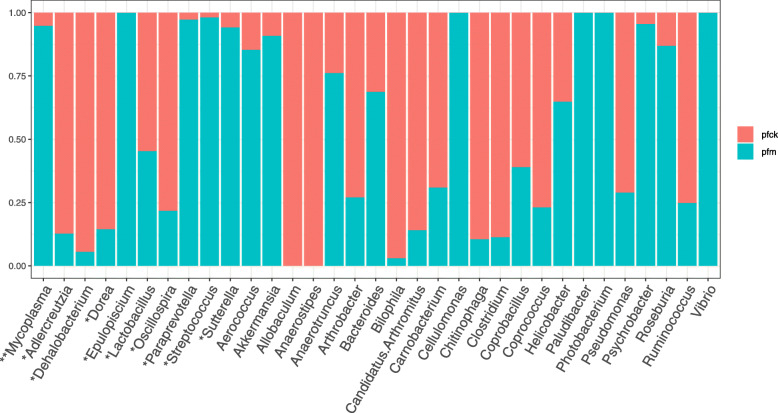
Dominant bacteria communities of mice intestinal microbiota of the two groups at the genus level. *, *q*-value < 0.05; **, *q*-value < 0.01

*Lactobacillus* had high abundance in pfck group (57%) and pfm group (69%) on phylum level. And as shown in Fig. 9, comparing the OTU abundance of the two groups, there were 33 bacterial microbiota communities (*p*-value < 0.05) with significant difference in OTU abundance between the two groups. Comparing the OTU abundance of the two groups, there were 10 bacterial microbiota communities with OTU abundance greater than 0.1% and *q*-value less than 0.05 on genus level. *Paraprevotella*, *Streptococcus*, *Epulopiscium, Sutterella* and *Mycoplasma* increased significantly in the genus level of intestinal microbiota in mice with *Folium sennae* intervention (*q*-value < 0.05); the abundance of *Adlercreutzia*, *Lactobacillus*, *Dehalobacterium*, *Dorea* and *Oscillospira* were reduced in the pfm group (*q*-value < 0.05).

#### VMH simulation of tryptophan metabolism in intestinal microbiota

Bacteria communities labeled by OTU which its abundance changed with the intervention of diarrhea caused by *Folium sennae* were selected to simulate tryptophan metabolism of intestinal bacteria. The intestinal metabolic function of the 10 microbiota from the above results was simulated, 7 of which were related to tryptophan metabolism through the synthesis of functional enzymes (Table. 2). In theory, all the microflora except *Paraprevotella* interfered by *Folium sennae* extracts were influence EX_trp_L(e) and TRPt2r to complete the metabolism of tryptophan, and L - Tryptophan was produced by the intestinal microbiota enzymatic reaction and completes amino tryptophan metabolism. We therefore concluded that diarrhea caused by *Folium sennae* influenced mainly tryptophan metabolism of intestinal microbiota.

**Table 2:** VMH simulation of tryptophan metabolism in intestinal flora

Bacteria	Reactions		Score	Metabolites	
	Abbreviation	Description		Abbreviation	Description
*Streptococcus*	5HLTDL	5-Hydroxy-L-Tryptophan Decarboxy-Lyase	4	Trp-L 5htrp	L-Tryptophan 5-Hydroxy-L-tryptophan
	LTDCL	L-Tryptophan Decarboxy-Lyase	4		
	5HXKYNDCL	5-Hydroxykynurenamine Decarboxy-Lyase	1		
	EX_5htrp(e)	Exchange of 5-Hydroxy-L-Tryptophan	1		
	EX_trp_L(e)	Exchange of L-Tryptophan	1		
	ACACT1r	Acetyl Coenzyme A C-Acetyltransferase	0		
	TRPt2r	L-tryptophan reversible transport via proton symport	0		
*Sutterella*	FKYNH	N-Formyl-L-Kynurenine Amidohydrolase	4	Trp-L	L-Tryptophan
	EX_trp_L(e)	Exchange of L-Tryptophan	1		
	TRPS1	Tryptophan synthase (indoleglycerol phosphate)	0		
	TRPS2	tryptophan synthase (indole)	0		
	TRPS3	tryptophan synthase (indoleglycerol phosphate)	0		
	TRPt2r	L-tryptophan reversible transport via proton symport	0		
*Dorea*	EX_trp_L(e)	Exchange of L-Tryptophan	1	Trp-L	L-Tryptophan
	ANPRT	Anthranilate phosphoribosyltransferase	0		
	ANS	Anthranilate synthase	0		
	ANS2	Anthranilate synthase 2	0		
	IGPS	Indole-3-glycerol-phosphate synthase	0		
	PRAI	Phosphoribosylanthranilate isomerase	0		
	TRPS1	Tryptophan synthase (indoleglycerol phosphate)	0		
	TRPS2	tryptophan synthase (indole)	0		
	TRPS3	tryptophan synthase (indoleglycerol phosphate)	0		
	TRPt2r	L-tryptophan reversible transport via proton symport	0		
	TRPTA	tryptophan transaminase	0		
*Lactobacillus*	EX_trp_L(e)	Exchange of L-Tryptophan	1	Trp-L	L-Tryptophan
	ACACT1r	Acetyl Coenzyme A C-Acetyltransferase	0		
	TRPt2r	L-tryptophan reversible transport via proton symport	0		
*Mycoplasma*	EX_trp_L(e)	Exchange of L-Tryptophan	1	Trp-L	L-Tryptophan
	TRPt2r	L-tryptophan reversible transport via proton symport	0		
*Adlercreutzia*	EX_trp_L(e)	Exchange of L-Tryptophan	1	Trp-L	L-Tryptophan
	TRPt2r	L-tryptophan reversible transport via proton symport	0		
*Paraprevotella*	ANPRT	Anthranilate phosphoribosyltransferase	0	Trp-L	L-Tryptophan
	ANS	Anthranilate synthase	0		
	ANS2	Anthranilate synthase 2	0		
	IGPS	Indole-3-glycerol-phosphate synthase	0		
	PRAI	Phosphoribosylanthranilate isomerase	0		
	TRPS1	Tryptophan synthase (indoleglycerol phosphate)	0		
	TRPS2	tryptophan synthase (indole)	0		
	TRPS3	tryptophan synthase (indoleglycerol phosphate)	0		
	TRPTA	tryptophan transaminase	0		

## Discussion

**Fig. 10:**
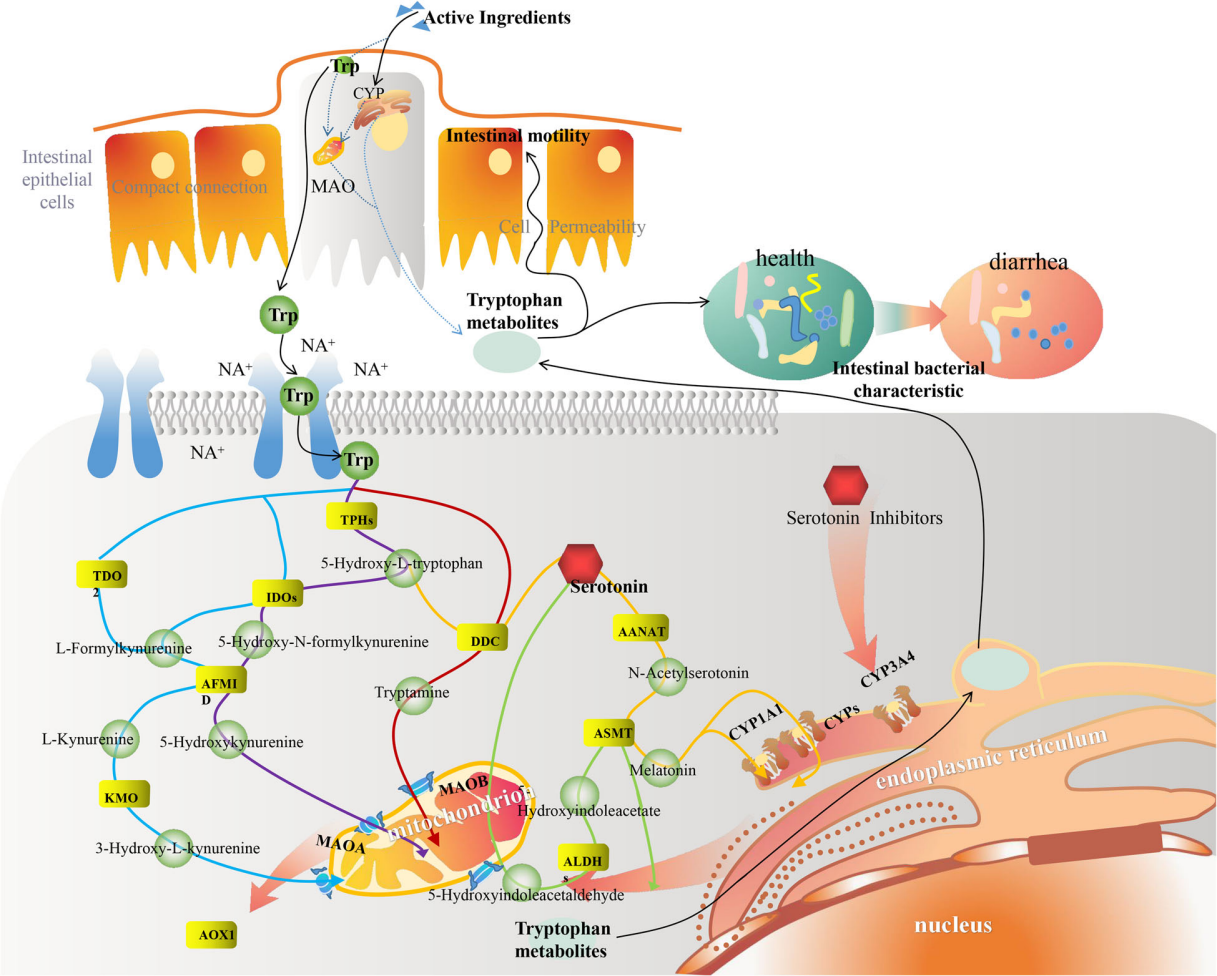
The intestinal microecological mechanism of diarrhea caused by *Folium sennae* extracts. According to the metabolic pathway of tryptophan enriched by KEGG in diarrhea caused by *Folium sennae*, we found that the *Folium sennae* extracts (Active Ingredients) can intervene MAOs by influencing CYPs. The metabolism of tryptophan by intestinal epithelial cells and its derivatives (Tryptophan metabolites) can alter intestinal bacterial characteristicthe, which leads to diarrhea

*Folium sennae*, as a ethnic Chinese traditional medicine, often causes diarrhea due to its laxative action. The dramatic impact of the commensal microbiota on the digestive disorders including diarrhea is increasing in appreciation. Although reports have provided evidence that *Folium sennae* can result in intestinal microbiota diversity disorder, the specific mechanism is still unknown for the gap of gaining knowledge that regarding the factors that drive different microbiota trajectories [21]. Therefore, this study was based on animal experiment and the techniques of metagenomics sequencing, drug target protein bioinformatics and microbial VMH intestinal simulation were utilized to probed the molecular mechanism of the variety of intestinal bacterial characteristic caused by *Folium sennae* diarrhea **(**Fig. 10**)**.

According to the results, diarrhea caused by *Folium sennae* altered intestinal bacterial characteristic and affected the diversity of intestinal microbiota in mice. The characteristics of community structure variety of intestinal microbiota caused by *Folium sennae*-induced diarrhea showed the decrease of the abundance of probiotics and the increase of the abundance of pathogenic bacteria. In our study, diarrhea intervened by *Folium sennae* was negatively correlated with *Lactobacillus* abundance. Select species of *Lactobacillus* is the most commonly included genus in modern probiotic supplements [22]. Although the purported health benefits of *Lactobacillus* probiotics vary significantly in different diseases, high-dose Lactobacillus preparation therapy at the early stage of diarrhea is recommended [23]. We also found that diarrhea intervened by *Folium sennae* increased the expression of pathogenic bacteria such as *Streptococcus* and *Mycoplasma* in intestinal tract of mice. *Streptococcus* and *Mycoplasma* in biofilm growth phase are principally responsible for an array of infections within hospital and bacteria in the biofilm state could resist host immune response and triggers infection [24]. The mice were given oral intranasal *Streptococcus pneumoniae* and induced antigen-specific mucosal and systemic immune responses, which is closely related to the severity of diarrhea [25]. Moreover, clinical case reported that patient infected with *Mycoplasma* without relevant past medical history was admitted to the hospital with diarrhea [26]. Accordingly, we believe that one of the ways to exert the pharmacological effect of *Folium sennae* on diarrhea is to regulate the structure of intestinal microbiota community.

We filtered AKT1, TNF and CYP3A4 as the main target protein genes of diarrhea caused by *Folium sennae* extracts by network pharmacology. As shown in Fig. 10, according to KEGG signaling pathway map, we found only CYP3A4 was involved in tryptophan metabolism. CYPs play a central role in the metabolism and elimination of xenobiotics including drugs, environmental pollutants, and food ingredients, and it is widely distributed in the liver and intestine of human body [27]. In both gut and liver, the functions of CYP enzymes 3A4 are determined by a complex interplay between genetic polymorphisms, the inductive or inhibitory effects of many drugs, herbs, food constituents and endogenous substances, and contributing to the decisive factors of clinical diarrhea [26]. CYPs plays an important role in drug metabolism and endogenous substance metabolism, and diarrhea might also occur [28]. The oxygenations of inert organic substrates by CYP450 would destroy nearby amino acids, as these reactions involve multiply bonded iron-oxos [29]. Peng et al. [30] found that tryptophan was soluble to CYP450 and Phosphatidylethanolamine (PE) decreased solvent accessibility for tryptophan in CYP450. Besides, previous studies have reported interactions of natural medicinal ingredients interactions can regulate tryptophan metabolism [31]. It is therefore rational to consider *Folium sennae* could regulate CYP3A4 and influence tryptophan metabolism, which is consistent with our results.

The relationship between tryptophan and diarrhea has been widely recognized, and a tryptophan hydroxylase inhibitor is increasingly used in the treatment paradigms of refractory diarrhea for the key role of 5 - Hydroxytryptophan (5 - HT) in tryptophan metabolism [32, 33]. Tryptophan hydroxylase 2 (TPH2) is the rate-limiting enzyme in 5-HT biosynthesis, and neuron production of 5-HT links intestinal dysfunction with tryptophan metabolism [34]. Intestinal microbiota exert anti-inflammatory properties facilitating the biosynthesis and fermentation of various amino acids including aminotryptophan in intestinal diseases [35]. Moreover, there’s research that CYP 450 transgenic mice were used to prove that regulation of intestinal and hepatic drug-processing enzymes by the intestinal microbiota via tryptophan and its metabolites [36]. Kaempferol, as one of the active ingredients in *Folium sennae* filtered in network pharmacology in this study, increased small bowel transit in mice through cholinergic pathways [37] and regulated the metabolites involved in tryptophan [38]. So we come to the conclusion that *Folium sennae* targeted CYP 3A4 to altered intestinal microbiota microecology and intervened the tryptophan metabolism of intestinal microbiota, which may be one of the pharmacological mechanisms of laxative action of *Folium sennae*.

## Conclusions

*Folium sennae* targeted cytochrome P450 (CYP) 3A4 to alter intestinal bacterial characteristic and to intervene the tryptophan metabolism of intestinal microbiota, such as *Streptococcus*, *Sutterella* and *Dorea*, which may be the intestinal microecological mechanism of diarrhea caused by *Folium sennae* extracts.

## Methods

### Experiment materials and reagents

*Folium sennae* used in this study was purchased from Xinglin Clinic of Hunan University of Chinese Medicine and originated in Yunnan Province. Specific pathogenfree Kunming mice were purchased from Hunan Slaccas Jingda Laboratory Animal Company. All animal work was carried out in accordance within the guidelines of the Institutional Animal Care and Use Committee of Hunan University of Chinese Medicine.

Referring to our previous experiment [39], 500 g *Folium sennae* was washed in cold water and naturally dried. The cleaned *Folium sennae* was soaked in 5 L boiling water for 10 min. The soak solution was filtered through 0.45 um filter membrane. Then the filtrate was evaporated and concentrated into 500 ml (1 g/mL crude drug) decoctioned in a 70 °C rotary evaporator and preserved at 4 °C.

### Animals and procedures

Twelve specific pathogen free Kunming mice (10 - wk. - old, 20 ± 2 g on average, male: female = 1: 1) were selected and fed in the laboratory for 2 days for adaptation, then were randomly divided into blank control group (Group pfck) and tested group (Group pfm), with 6 mice (3 male and 3 female) in each group. The mice in Group pfm were used for intragastric administration in *Folium sennae* extracts per treatment. Kept in separated cages in a clean and quiet environment with a temperature of 23–25 °C and humidity of 50–70%, all the mice are bred in standard particles.

*Folium sennae* extracts prepared according to step 1 were given by gavage to the mice in Group pfm at 9:00 am and 16:00 pm at a dosage of 0.4 ml/1 for 7 days continuously. Sterile saline of the same volume was given to the mice by means of intragastric administration in Group pfck twice a day in the continuous 7 days. During intragastric administration of animals process, the weight and defecation behavior of mice was recorded every other day. Mice in 2 groups (Group pfck and pfm) were sacrificed by cervical dislocation at day 8, and the contents of the intestine were collected under sterile conditions and preserved at − 80 °C [40].

### Analysis of bacterial characteristic in intestinal contents

#### Total DNA extraction from intestinal contents

Mice were euthanized by cervical dislocation. On the clean bench, the abdomen of the mouse was cut open along the abdomen white line to expose the ileum and colorectal area. The intestinal tract was dissected with sterile scissors, and then intestinal contents were collected using a sterile device. According to our previous studies [39, 40], for the differences in the composition of intestinal microbiota caused by the specificity of sex, we controlled the quality of samples in mixing sexes. In this study each sample was contained intestinal contents derived from two mice (one male and one female) for the total DNA extraction. 0.2 g of intestinal contents were weighed and suspended with 0.1 mol·L-1 phosphate buffer solution (PBS) to remove the food residue. Subsequently, all the supernatants were transferred into fresh tubes and centrifuged at 12,000 rpm/min at low temperature for 5 min to obtain intestinal microorganisms. According to the protocols previously reported, total intestinal microbial DNA was eventually dissolved in 50 μL TE buffer for further analysis after testing with 1.5% agarose gel electrophoresis.

#### PCR amplification and sequencing

The V3-V4 hypervariable regions of the bacteria 16S rRNA gene were amplified with primers 338F (5′- ACTCCTACGGGAGGCAGCAG-3′) and 806R (5′-GGACTACHVGGGTWTCTAAT-3′) by thermocycler PCR system (GeneAmp 9700, ABI, USA). The PCR reactions were conducted using the following program: PCR mixture (25 μL) included 5 μL 5 × reaction buffer, 5 μL 5 × high GC buffer, 1 μL 10 μmol/L forward primer, 1 μL 10 μ mol/L reverse primer, 0.25 μL Q5 high-fidelity DNA polymerase, 0.5 μL 10 mmol/L dNTP, 1 μL DNA template and 11.25 μL sterilized ddH_2_O. The PCR conditions were as follows: initial denaturation at 98 °C for 30 s; denaturation at 98 °C for 15 s, annealing at 46 °C for 30 s and extension at 72 °C for 30 s, repeated for 32 cycles; last cycle of final extension at 72 °C for 5 min and holding at 4 °C. PCR products were detected by 2% Agarose gel electrophoresis. Libraries were prepared using TruSeq DNA LT Sample Preparation Kits (Illumina) and sequenced by Illumina MiSeq using the MiSeq Reagent Kit.

### Identify of potential active components, gene targets and metabolic mechanisms

#### Database construction and datasets analysis

To construct chemical ingredients of *Folium sennae* database, information of chemical compounds of *Folium sennae* was obtained from Traditional Chinese Medicine Systems Pharmacology Database and Analysis Platform (TCMSP, http://lsp.nwu.edu.cn/tcmsp.php). The potential active components that satisfying both oral bioavailability (OB) ≥ 30% and drug-likeness (DL) ≥ 0.18 of *Folium sennae* were selected [20]. The target prediction for the main active compounds screened out by the above step was performed using the PharmMapper server (http://www.lilabecust.cn/pharmmapper/) with the “*Homo sapiens*” species setting, and the gene targets of *Folium sennae* were selected. After searching for Genecard datasets (https://www.genecards.org/) using keywords “diarrhea” [41], all gene targets of diarrhea disease were enrolled. In the next step, the two datasets of gene targets are intersected to identify gene targets of *Folium sennae* for diarrhea disease. The target genes were selected, and gene information including gene ID, name, and organism was identified using UniProt database (https://www.uniprot.org/).

#### Construction of network

To identify the functional protein of the gene targets of *Folium sennae* for diarrhea disease, the PPI network was constructed using the STRING online database (https://www.string-db.org/). In order to understand the mechanisms of *Folium sennae* for diarrhea disease, Drug-Disease-Target network analysis was performed. The corresponding network was established and visualized by Cytoscape 3.7.1 software.

#### Analysis of the pathway and functional enrichment

The KEGG is a reference resource for biological interpretation of high-throughput genes and protein pathways. We carried out KEGG functional and signaling pathway enrichment analysis for predicting targets associated with metabolism of *Folium sennae* for diarrhea disease by using DAVID bioinformatics resources 6.8 (https://david.ncifcrf.gov/).

### VMH virtual machine to simulated metabolic function of gut microbiome

The enteric microorganisms in the intestinal contents samples of mice were measured using a metagenomics method. The V3 ~ V4 region of DNA was amplified by PCR, and then using Qiime platform 1.7.0 (http://qiime.org/) to cluster 97% similarity DNA sequences into a same OTU. Comparing Greengene database to annotate the taxonomic information of each OTU. At the level of microbial taxonomy, analysis of the effect of diarrhea caused by *Folium sennae* on the OTU abundance of intestinal microbial colonies.

The Virtual Metabolic Human (VMH, www.vmh.life) represents a novel, interdisciplinary database for data interpretation and hypothesis generation to the biomedical community [42]. The VMH database encapsulating current knowledge of human metabolism within ‘Human metabolism’, ‘Gut microbiome’ and ‘Disease’ interlinked resources. In this study, metabolism of the intestinal microbial colonies with significant changes in OTU abundance was simulated by VMH virtual machine to verify the metabolites and metabolic pathways derived from KEGG enrichment analysis.

### Statistical analysis

Statistical analyses were performed with GraphPad Prism 8.0 and R 3.6.1 statistical software using the Student’s paired or unpaired t test, and Chi-square test. *p*-value < 0.05 was considered as statistically significance.

## Supplementary information

**Additional file 1 **Supplementary **Table 1**. Active substances and target genes related to diarrhea in *Folium sennae*.

**Additional file 2 **Supplementary **Table 2**. Disease score of diarrhea-related target genes in *Folium sennae*.

## Data Availability

The datasets used and analysed during the current study are available from the corresponding author on reasonable request.

## Abbreviations

AKT1: Protein kinase B 1
CYP450: Cytochrome P450
CYP3A4: Cytochrome P450 3A4
DL: Drug-likeness
5-HT: 5-Hydroxytryptophan
KEGG: Kyoto Encyclopedia of Genes and Genomes
OB: Oral bioavailability
OTU: Operational taxonomic unit
PCA: Principal Component Analysis
PBS: Phosphate buffer solution
PPI: Network protein protein interaction netmork
TCMSP: Traditional Chinese Medicine Systems Pharmacology Database and Analysis Platform
TNF: Tumor Necrosis Factor
TPH2: Tryptophan hydroxylase 2
VMH: Virtual Metabolic Human
